# Adjuvant Use of Ivabradine in Acute Heart Failure due to Myocarditis

**DOI:** 10.1155/2011/203690

**Published:** 2011-09-27

**Authors:** Jennifer Franke, Dorothee Schmahl, Stephanie Lehrke, Regina Pribe, Raffi Bekeredjian, Andreas O. Doesch, Philipp Ehlermann, Philipp Schnabel, Hugo A. Katus, Christian Zugck

**Affiliations:** ^1^Department of Cardiology, University of Heidelberg, Im Neuenheimer Feld 410, 69120 Heidelberg, Germany; ^2^Institute of Pathology, University of Heidelberg, 69120 Heidelberg, Germany

## Abstract

We report two cases of young men in whom acute heart failure due to myocarditis was diagnosed. The patients had been transferred to the intensive care unit (ICU) with commencing symptoms of acute heart failure and consecutive multiorgan failure for further treatment and to evaluate the indication for implantation of a ventricular assist device or for high urgent orthotopic heart transplantation. In both patients, the *I*_*f*_-channel inhibitor ivabradine was administered off-label to provide selective heart rate reduction, and thus support hemodynamic stabilization. Though currently considered off-label use in patients suffering from severe hypotension and acute heart failure, the use of ivabradine may beneficially influence outcome by allowing optimization of the patient's heart rate concomitant to initial measures of clinical stabilization.

## 1. Introduction

Although the energy-efficient mechanisms of heart rate reduction are apparent, in the event of acute heart failure due to myocarditis, the utilization of this therapeutic strategy has only played a minor role. Due to the hypotensive character of beta-receptor-blockers and class IV antiarrhythmics, physicians have been constrained to administering these agents subsequent to stabilizing the patient and not in the advent of acute distress [1]. Ivabradine is a selective heart-rate-lowering agent that acts on the sinoatrial node without the undesired effect of hypotension. By inhibiting the activity of the *I*_*f*_ channel, ivabradine decelerates the gradient of diastolic depolarisation and by this reduces the intrinsic pacemaker activity in the sinoatrial node. Due to this unique mechanism of heart rate reduction without influence on myocardial contractility, it is conceivable that ivabradine may also play a key role in the therapy of acute heart failure.

## 2. Case Presentation

We report two cases of young men in whom acute heart failure due to myocarditis was diagnosed. The patients had been transferred to the intensive care unit (ICU) of our department with commencing symptoms of acute heart failure and consecutive multiorgan failure for further treatment and to evaluate the indication for implantation of a ventricular assist device or for high urgent orthotopic heart transplantation. In both patients, the *I*_*f*_-channel inhibitor ivabradine was administered off-label to provide selective heart rate reduction, and thus support hemodynamic stabilization.

**Figure 1:**
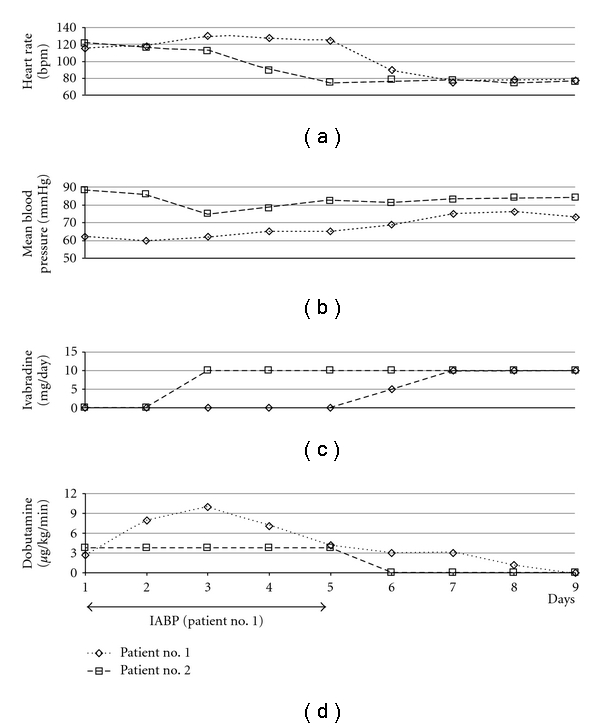
Therapy and hemodynamic parameters of patient no. 1 and no. 2 throughout the first nine days of intensive care treatment. IABP: intra-aortic balloon counterpulsation.

**Figure 2:**
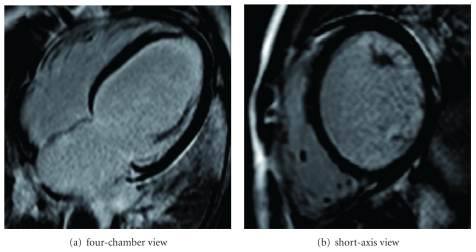
Epicardial late enhancement in the basal inferolateral region in cardiac MRI.

Patient no. 1, a previously healthy 18-year-old man, was transferred to the ICU of our department of cardiology from a secondary hospital for further treatment. Despite all attempts to stabilize the patient at the prior hospital, including therapy with inotropics, levosimendan, and high doses of furosemide i.v., clinical symptoms of right and left heart failure had significantly worsened within days. Recent clinical history indicated an onset of flu-like symptoms including dry cough and fever continuing for seven days followed by progression of dyspnoea, which led to the initial hospital admission ten days before. At the time of transfer to our ICU, the patient suffered acute heart failure with subsequent acute renal failure and severe liver and pulmonary congestion. The patient's mean heart rate was 115 bpm, and mean blood pressure was 62 (85/50) mmHg (Figure 1). Cardiac catheterization showed normal coronary arteries and a globally dilated heart with severely impaired systolic left ventricular (LV) function. LV ejection fraction measured 20% in echocardiography. The patient rejected a myocardial biopsy under the aspect of the elevated risk of bleeding subsequent to his liver congestion; therefore, histological examinations were not possible. Although cardiac MRI ruled out myocardial edema, epicardial late enhancement was detected in the basal inferolateral region which was interpreted as an unspecific postmyocarditis phenomenon (Figure 2). As further circulatory support was required, intra-aortic balloon counterpulsation (IABP) was initiated, and inotropic treatment with dobutamine was increased to 10 *μ*g/kg/min. Cardiac output measured 4.1 L/min initially and raised to a maximum of 6.0 L/min (stroke volume 46.2 mL/beat) under increased dobutamine therapy. Though the patient was successfully weaned from IABP after five days, the dobutamine dose could not be reduced and consecutively led to sinus tachycardia (mean heart rate 130 bpm). After receiving approval of the institutional review body and the patient's informed consent, the *I*_*f*_-channel inhibitor ivabradine was administered off-label to provide selective heart rate reduction. Ivabradine therapy was initiated with 5 mg twice daily orally. One day after ivabradine therapy was started, the patient's heart rate reduced significantly to 75 bpm, and blood pressure increased to 110/60 mmHg. Concordant to the prompt improvement in hemodynamic parameters, an improvement of diuresis was achieved. Over the following three days, the patient was successfully weaned from catecholamines, and the patient's body weight markedly reduced. Invasive measurements revealed a cardiac output of 5.8 L/min (stroke volume 72.5 mL/beat). In echocardiography, left ventricular ejection fraction increased minimally from 20 to 25%. In addition, we observed a lowering of N-terminal probrain natriuretic peptide (NTproBNP)—from 6237 ng/L to 3920 ng/L, which corresponded well with the noticeable clinical improvements. After discharge, the patient underwent close clinical followup at our specialized outpatient heart failure clinic. After gradual uptitration of a beta-receptor-blocker (carvedilol), ivabradine was reduced after approximately 9 months (274 days) without complications. As of this writing, clinical followup of patient no. 1 is available up to 463 days after initiation of ivabradine. During this last outpatient visit, the patient showed no functional limitations (NYHA I), and his NTproBNP reduced to a nearly normal value of 192 ng/L (age-dependant normal values are below 125 ng/L).

**Figure 3:**
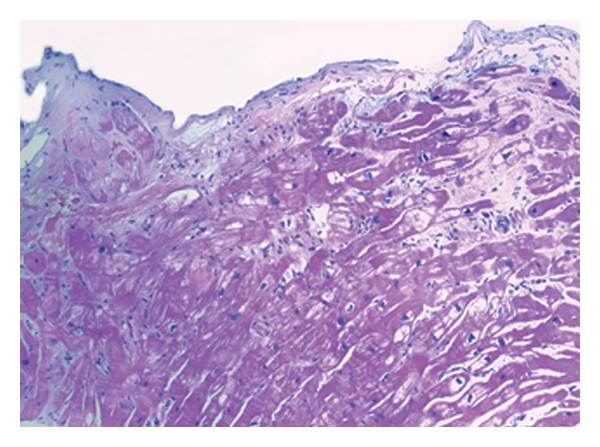
Inflammatory cardiomyopathy with mononuclear infiltration in histopathological analysis.

Patient no. 2, a previously healthy 28-year-old man, was transferred to our ICU by a collaborating tertiary hospital to evaluate the indication for implantation of a ventricular assist device or for high urgent orthotopic heart transplantation. At arrival, the patient presented commencing symptoms of acute heart failure and consecutive multiorgan failure, including mild pancreatitis, acute renal failure requiring daily dialysis, and severe liver and pulmonary congestion. His mean heart rate was 120 bpm. In spite of previous treatment with inotropics and levosimendan over five days, clinical stabilization had so far been unsuccessful. The patient's recent clinical history revealed flu-like symptoms including cough, diarrhoea, and fever which began one week prior to his initial admission. His chest X-ray showed no signs of pneumonia, and microbiological tests were negative. In echocardiographic evaluation, an ejection fraction of 10% and an increased left ventricular enddiastolic diameter of 75 mm were measured (body-surface-adapted normal values for men are below 56 mm). Cardiac catheterization ruled out coronary heart disease. Multiple endomyocardial biopsies taken during the procedure confirmed the diagnosis of inflammatory cardiomyopathy with mononuclear infiltration in histological analysis (Figure 3). As in the case of patient no. 1, weaning from inotropic support (dobutamine 3.7 *μ*g/kg/min) was primarily unsuccessful, and sinus tachycardia remained a relevant clinical symptom (Figure 1). The patient gave informed consent to an off-label therapy with ivabradine to treat sinus tachycardia selectively. Within 24 hours, after we initiated ivabradine therapy (5 mg twice daily orally), the patient's heart rate had lowered to 89 bmp and further to 75 bpm in the following day. In addition to the noticeable heart rate reduction within 48 hours, the patient's blood pressure stabilized at 120/65 mmHg, and inotropic therapy with dobutamine could be discontinued. Invasive measurements to calculate cardiac output were not performed in this patient. Repeat echocardiography after the initiation of ivabradine revealed a consistent left ventricular ejection fraction of 10%. Sequential measurements of NTproBNP taken shortly after admission, then after clinical improvement, demonstrated a reduction of NTproBNP value from 81,195 ng/L to 28,161 ng/L. Ivabradine therapy was discontinued, and beta-receptor-blocker therapy was gradually uptitrated after approximately 1 month (35 days) without clinical complications. During the follow-up period of 232 days, the patient further recovered and currently has no functional limitations (NYHA I). NTproBNP reduced to 183 ng/L.

## 3. Discussion

The range of indications for ivabradine includes treatment of stable angina pectoris in patients with sinus rhythm and, in conjunction to beta-receptor-blockers, treatment of patients with chronic heart failure [2, 3]. Of the relatively few adverse drug reactions described in the two largest randomized trials which evaluated ivabradine in these cohorts, transient phosphenes (visual sensations of enhanced brightness) and bradycardia accounted for the most frequent drug-associated complications. Fortunately, these adverse drug effects only rarely led to discontinuation of pharmacotherapy. In the absence of evidence from other, more far-reaching clinical trials, ivabradine is currently contraindicated in patients suffering from severe hypotension and/or acute heart failure. However, it is conceivable that this novel agent may be advantageous through more than one mechanism in exactly these specific situations, first by counteracting dobutamine-induced sinus tachycardia, which frequently limits the success of stabilization in the case of acute heart failure. In contrast to beta-receptor-blockers or class IV antiarrhythmics such as Diltiazem or Verapamil, ivabradine does not share their hypotensive character which largely limits their administration in the advent of acute heart failure. Secondly, although substantial clinical evidence is pending, experience from one preclinical study suggest that ivabradine may play a modulating role in perivascular and interstitial inflammatory reactions in acute myocarditis and in myocardial infarction [4]. 

A similar case of acute heart failure and dobutamine-induced sinus tachycardia has previously been reported. The authors described beneficial hemodynamic effects in a patient with severe ischemic cardiomyopathy. The patient's heart rate decreased from 114 to 75 bpm after initiation of ivabradine, and a discrete increase in stroke volume was noticed [5]. Others have described the use of ivabradine in a patient with acute myocardial infarction and cardiogenic shock. Despite successful recanalization of the left main coronary artery, the patient's heart rate was 120 bpm. Weaning of IABP and inotropic medical treatment (drug not specified) was not possible due to severe hypotension. Forty-eight hours after introducing ivabradine to the drug regime, heart rate reduced to 80 bpm, and the patient was successfully weaned from IABP and inotropics [6]. Mulder et al. were the first to demonstrate preservation of cardiac output despite the decrease in heart rate after administration of ivabradine in a rat model of chronic heart failure. The authors hypothesized that prolongation of the diastolic phase through the administration of ivabradine increases left ventricular filling, and thus enhances stroke volume despite heart rate reduction [7]. In a corresponding clinical study, hemodynamic effects of ivabradine were evaluated in ten patients with advanced heart failure (NYHA III). The authors reported an increase of stroke volume of up to 51% after initiation of ivabradine therapy. Similarly, we detected an increase of stroke volume (+36%) in patient no. 1 despite heart rate reduction and simultaneous weaning of inotropic support [8].

Within the limitations of data obtained from just two patients, this present experience can only be hypothesis generating. We suggest that selective heart rate reduction with ivabradine may beneficially influence the outcome of patients presenting with the above-mentioned symptoms in the course of myocarditis by allowing optimization of the patient's heart rate concomitant to initial measures of clinical stabilization.
